# Supplementary material for: A note on variable susceptibility, the herd-immunity threshold and modeling of infectious diseases
Source: PLoS One. 2023 Feb 15;18(2):e0279454. doi: 10.1371/journal.pone.0279454 (PMC9931097; doi:10.1371/journal.pone.0279454)
Supplement: S1 File — (PDF) [file pone.0279454.s002.pdf]

Supplementary Material

1 Modeling of variable susceptibility and infectivity

We first build a compartmental model taking variable infectivity and susceptibility into account, and then prove that variable infectivity is irrelevant, under the assumption that the two are not correlated (otherwise the statement is false, see e.g. [1]). This is not surprising, see e.g. [2], but it is still worthwhile to prove within our modeling framework. Section 2 is the key contribution where we prove that the resulting susceptibility-stratified model can be further reduced to a basic SIR with ASI.

When modeling variable infectivity and super-spreaders, it is important to distinguish between super-spreaders in the sense that they have an extremely high viral load (possibly combined with lack of symptoms [3, 4]), and *social* super-spreaders, which are persons whose work or behavior puts them in contact with an unusually high number of people. The latter type was discussed in Section 4.2, so we focus here on the former type. We refer to [5] and [6] for the basics of mathematical modeling of infectious diseases.

Let the susceptibles be divided into J subgroups, and let the infectives be divided into K subgroups. Let $\mathbf{S} = (S_1, \ldots, S_J)^T$ be a vector such that $S = \sum_{j=1}^{J} S_j$ is the total amount of susceptibles, where the symbol T denotes transpose, i.e. the operation that turns a row-vector into a column-vector. (All vectors considered here are to be column-vectors for the matrix vector formalism to function.) Similarly $\mathbf{I} = (I_1, \ldots, I_K)^T$ will represent the various compartments of I where $I = \sum_{k=1}^{K} I_k$. All the above quantities implicitly depend also on time t which is a continuous variable. Hence $S(t)$ and $I(t)$ denotes the total amount of susceptibles and infectives at a given time t, whereas $\mathbf{I}(t)$ and $\mathbf{S}(t)$ contain the amount in each respective compartment. Note that there is no need to introduce various compartments for R, since once you recover neither infectivity level nor susceptibility level is important. Let a be the daily amount of potentially infectious contacts by an average infective during his period of illness and let $p_{j,k}$ be the probability that an infectious encounter between a member of I_k and one in S_j leads to transmission, and let M_P denote the corresponding matrix. The rate of new infections in group S_j is then easily seen to be $\nu_j(t) = \frac{S_j(t)}{N} \sum_{k=1}^{K} p_{j,k} a I_k(t)$, or in

vector form

$$\boldsymbol{\nu} = \frac{a}{N}\text{diag}_{\mathbf{S}} M_P \mathbf{I} \tag{11}$$

where $\text{diag}_{\mathbf{S}}$ denotes the diagonal matrix with $\mathbf{S}(t)$ on the diagonal and $\boldsymbol{\nu}$ denotes $(\nu_1, \ldots, \nu_J)^T$. Since we assume that susceptibility and infectivity are uncorrelated, the total amount of new infections are distributed over the various I_k's in proportion to the fraction of the population that belong to respective group. More precisely, let x_k denote the fraction of the total population that would end up in infectivity group I_k, if falling ill, and let $\mathbf{x} = (x_1, \ldots, x_K)^T$ denote the corresponding vector. The corresponding SIR-equation system becomes

$$\begin{cases} \mathbf{S}' & = -\boldsymbol{\nu} \\ \mathbf{I}' & = \nu\mathbf{x} - \sigma\mathbf{I} \\ R' & = \sigma I(t) \end{cases} \tag{12}$$

where $\nu = \sum_{j=1}^{J} \nu_j$ (so it is not a misprint that one is bold and the other not). The initial conditions become

$$\begin{cases} \mathbf{S}(0) & = \mathbf{w}N \\ \mathbf{I}(0) & = \tilde{\mathbf{x}}n \\ R(0) & = 0 \end{cases} \tag{13}$$

where $\mathbf{w} = (w_1, \ldots, w_J)^T$ is the fraction of the population in the respective susceptibility group at the onset of the pandemic, n is the total number of import cases to the population of N individuals, and $\tilde{\mathbf{x}}$ a vector containing the fraction of these in each subgroup of I.

These are the equations we propose for disease spread in a model with variable (uncorrelated) susceptibility and infectivity. A weak point of such a model is clearly the difficulty of measuring reasonable values of the transmission probabilities $p_{j,k}$ in practice. However, this is not needed since the main point of this paper is that the output of any such model is almost identical to the output of the most basic possible SIR if we include ASI. Given a fixed value of σ (i.e. the inverse of the generation time $T_{generation}$), the basic SIR then depends only on two parameters, ASI θ and transmission rate α (or, equivalently, R_0). Hence, as long as we believe that there exist numbers such that the above system (11)-(13) would be a good model for accurately

forecasting disease spread, we can instead run a basic SIR-model with only two unknown parameters, which are (more) easily fitted to real data. In conclusion, we do not need to know the actual transmission probabilities $p_{j,k}$, or the contact rate a. We now begin to reduce these equation to simpler ones.

1.1 The initial conditions are irrelevant

Even if $\tilde{\mathbf{x}}$ is different from $\mathbf{x}$, it will not remain so for long, for the equation for $\mathbf{I}'$ in (12) ensures that at any time point the new cases are distributed according to $\mathbf{x}$. Hence it will only take a few generations before the distribution of infected very closely resembles that of $\mathbf{x}$. This will happen in the very early stages of the disease progression, and therefore we might as well assume that $\tilde{\mathbf{x}} = \mathbf{x}$ from the beginning.

More rigorously, we can argue as follows; Given the function ν describing the total incidence, the solution for I_j is given by

$$I_j(t) = x_j \int_0^t e^{\sigma(s-t)} \nu(s)\ ds + \tilde{x}_j e^{-\sigma t},$$

by which it follows that $\tilde{x}_j$ soon becomes irrelevant, due to the exponential decay of the corresponding term $\tilde{x}_j e^{-\sigma t}$.

1.2 Variable infectivity is irrelevant; S-SIR

We now reduce the above equations to the Susceptibility-stratified model (S-SIR) used in the main text. Recall that $\mathbf{I}$ denotes the vector of all infectivity compartments and I denotes their sum.

Proposition 1.1. *Given any solution* $(\mathbf{S}, \mathbf{I}, R)$ *to the system* (11)-(13) *with* $\tilde{\mathbf{x}} = \mathbf{x}$*, the triple* $(\mathbf{S}, I, R)$ *solves the system*

$$\boldsymbol{\nu} = \frac{a}{N}\mathbf{p} \cdot \mathbf{S} I \tag{14}$$

$$\begin{cases} \mathbf{S}' &= -\boldsymbol{\nu} \\ I' &= \nu - \sigma I \\ R' &= \sigma I \end{cases} \tag{15}$$

$$
\begin{cases} \mathbf{S}(0) &= \mathbf{w}N \\ I(0) &= n \\ R(0) &= 0 \end{cases} \tag{16}
$$

where $\cdot$ denotes componentwise multiplication, $\nu = \sum_{j=1}^{J} \nu_j$ and $\mathbf{p} = M_P\mathbf{x}$.

Proof. Let $(\mathbf{S}, \mathbf{I}, R)$ be given and let $\boldsymbol{\nu}$ be defined by (11). Given a scalar solution to the equation $i' = \nu - \sigma i$, $i(0) = n$, it follows that $I_j(t) = x_j i(t)$ is a solution to the corresponding row in the equation $\mathbf{I}' = \nu\mathbf{x} - \sigma\mathbf{I}$. By the uniqueness of such solutions, we conclude that $\mathbf{I}(t) = \mathbf{x}i(t)$. Hence, since I denotes the sum of $\mathbf{I}$, we see that $i = I$. With $\mathbf{p} = M_P\mathbf{x}$ it is easy to see that (11) reduces to (14), and that the equation for $\mathbf{I}$ in (12) reduces to the middle equation of (15).

□

Note that the numbers $p_1, \ldots, p_J$ in $\mathbf{p}$ are between 0 and 1 and can be interpreted as the probability of disease transmission when an "average" infective meets a susceptible in group $S_1, \ldots, S_J$, respectively. We can also compute R_0 given these transmission probabilities via the formula

$$
R_0 = a\mathbf{w}^T M_P \mathbf{x} T_{generation} = a\langle \mathbf{w}, \mathbf{p} \rangle T_{generation}, \tag{17}
$$

which should be compared with $R_0 = \alpha T_{generation}$ for the basic SIR (see Section 3.1). The simplest way to derive this is to note that at $t = 0$ we have $\mathbf{S}/N = \mathbf{w}$ so the equation for I then reads $I'(0) = a\langle \mathbf{w}, \mathbf{p} \rangle I - \sigma I$. This means that an average infective infects $a\langle \mathbf{w}, \mathbf{p} \rangle$ new individuals every day, which in average goes on for $T_{generation}$ amount of days. Multiplying these two quantities gives (17). The same formula can also be deduced more rigorously using the formal mathematical definition of R_0 as the spectral radius of the next-generation matrix, see e.g. Sec. 5 in [7].

In the S-SIR model (14)-(16), herd-immunity arise when the derivative $I'(t)$ becomes negative, i.e. at the point t_0 when the equation $a\langle \frac{\mathbf{S}(t_0)}{N}, \mathbf{p} \rangle - \sigma = 0$. Since it is not possible to theoretically compute the distribution of susceptibles at this point, we can not give a closed formula for the herd-immunity threshold. This underlines the

value of the result in the coming section, implying that the value is approximately

$$H_{IT} \approx \frac{\left(\sum_{j=1}^{J} w_j p_j\right)^2}{\sum_{j=1}^{J} w_j p_j^2}(1 - 1/R_0)$$

(obtained by combining (2) and (8)). The fact that the value is lower than the classical formula (1) is clearly related to the fact that highly susceptible individuals get infected to a greater extent than the other compartments. However, during a vaccination campaign there will be an equal fraction of immunity in in each respective group, i.e. a constant times $\mathbf{w}$. Letting $H_{IT,vac}$ denote the vaccine induced herd immunity threshold, we thus get the equation

$$a\langle(1 - H_{IT,vac})\mathbf{w}, \mathbf{p}\rangle - \sigma = 0$$

which in combination with (17) implies the classical formula $H_{IT,vac} = 1 - 1/R_0$.

1.3 Examples with variable susceptibility

To illustrate, we note that if $J = 3$, then (14) becomes

$$\begin{bmatrix} \nu_1(t) \\ \nu_2(t) \\ \nu_3(t) \end{bmatrix} = \begin{bmatrix} p_1 S_1(t) \\ p_2 S_2(t) \\ p_3 S_3(t) \end{bmatrix} \frac{aI(t)}{N} \tag{18}$$

and now the total amount of newly infected becomes

$$\nu(t) = \nu_1(t) + \nu_2(t) + \nu_3(t) = \frac{p_1 S_1(t) + p_2 S_2(t) + p_3 S_3(t)}{N} aI(t). \tag{19}$$

To see why this can not be further reduced to a basic SIR, in a similar way as the proof of Proposition 1.1, note that ν_1 gets withdrawn from S_1, ν_2 gets withdrawn from S_2 and ν_3 gets withdrawn from S_3. If individuals in S_1 are much more susceptible than individuals in S_2, who are much more susceptible than those in S_3 (so $p_1 \gg p_2 \gg p_3$) then the fraction $\nu_1(t)/S_1(t)$ is much larger than $\nu_2(t)/S_2(t)$ and $\nu_3(t)/S_3(t)$, which means that the group of "super-susceptibles" S_1 will become depleted faster. But if $p_1 \gg p_2 \gg p_3$ then the super-susceptibles contribute to the main part of the coefficient

in front of $I(t)$ in (19), and hence it turns out that the disease starts to recede when S_1 becomes sufficiently small, way ahead of what is expected from the standard (homogenous) SIR-model. In other words, while a standard SIR model needs to get above the classical herd-immunity threshold $1 - 1/R_0$ before it starts to die out, the model here can die out as soon as the super-susceptibles are depleted. Thus, once the super-susceptibles are depleted, the disease can die out by itself at fractions much lower than predicted by the classical HIT.

This phenomenon is clearly visible in Figure 2 in the main text (dashed curves). For that experiment, we assumed that S_1 consists of the super-susceptibles, for which $p_1 = 1$, and further that these constitute 30%, i.e. $w_1 = 0.3$. Similarly we assumed that the average group S_2 constitutes 60% and that only one in 10 contacts leads to disease transmission, i.e. $p_2 = 0.1$. Finally we supposed that the remaining 10% are well-protected with $p_3 = 0.02$. Clearly, the group of super-suseptibles have a huge attack rate of around 80% (compared with 72% for the whole population in the standard SIR using the same $R_0 = 1.66$), but as a consequence, the other two groups have attack rates of around 15% and 5%, respectively. This illustrates why traditional estimates for Herd-Immunity Threshold may be overly pessimistic.

To underline that the behavior of S-SIR seen in Figure 1 and 2 is representative, we provide a second example with only 10% super-spreaders and 30% well protected (i.e. we flip the amount in group S_1 with the amount in group S_3). The result is displayed in Supp. Fig. 1. As is plain to see, not much changes; the tiny group of super-susceptibles still "protects" the rest, in the sense that once they are depleted the disease dies out naturally. This group now has a 92% attack rate, as opposed to previously 80% in Fig. 2. Looking at the graph of recovered, we also see that it looks almost as either of the curves in Fig. 1a, only the final size of the pandemic changes to 23%, from 33% for the previous S-SIR and 72% for standard SIR, respectively. Intuitively, it should be possible to find a setting of parameters where the disease burns out among the super-susceptibles, but lives on a bit longer in the "average" group. However, the main finding of this paper is that this intuition is wrong, as we establish in the next section.

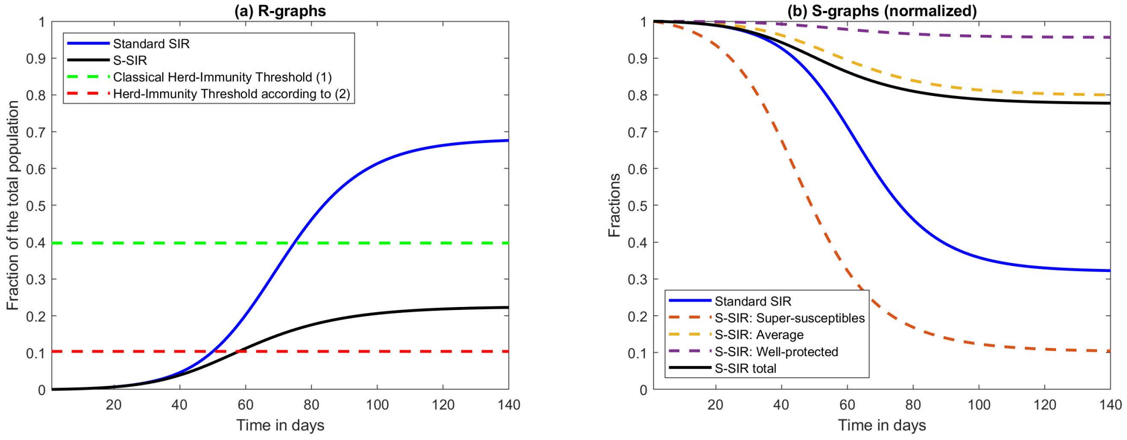

Supp. Fig 1. Comparison graphs of R and S for alternative compartment sizes. (a) Graphs of recovered as fraction of the population for SIR and S-SIR with a fixed value of $R_0 = 1.66$, along with estimates for the Herd-Immunity Threshold. As expected, S-SIR reaches a final size of the pandemic way below the classical HIT, but about twice that of the HIT as computed according to formula (2) suggested in this paper. (b) Corresponding graphs for susceptibles, which should be compared with Figure 2 in the main text. The model assumes 10% super-spreaders and 30% well protected, as opposed to the situation with 30% super-spreaders and 10% well protected depicted in Figure 2.

2 Variable susceptibility is almost equivalent to ASI

We now motivate the claims made in Section 3 in the more general setting of the Susceptibility-stratified SIR (14)-(16). We first introduce some notation and explain the intuition behind the formula (8). First consider standard SIR (3)-(5), and let (S_0, I_0, R_0) be a particular solution. We introduce the normalized functions $s_0(t) = S_0(t)/N$, $i_0(t) = I_0(t)/N$, $r_0(t) = R_0(t)/N$. These functions solve the system

$$\begin{cases} s' &= -\alpha s i \\ i' &= \alpha s i - \sigma i \\ r' &= \sigma i \end{cases} \tag{20}$$

with $s(0) = \omega$, $i(0) = n/N =: \varepsilon$ and $r(0) = 0$. Note that $s_0(t) + i_0(t) + r_0(t) = \omega + \varepsilon$. Next, we reduce (20) by using the well-known fact that t can be replaced by r as the independent variable. Let $\tilde{s}_0$ and $\tilde{i}_0$ be functions defined via $s_0(t) = \tilde{s}_0(r_0(t))$ and $i_0(t) = \tilde{i}_0(r_0(t))$. By the chain rule the functions $(\tilde{s}_0, \tilde{i}_0)$ are solutions to the system

$$\begin{cases} ds/dr &= -\alpha s/\sigma \\ di/dr &= \alpha s/\sigma - 1 \end{cases} \tag{21}$$

which is easily solved. Since $r_0(0) = 0$ we have that $\tilde{s}_0(0) = s_0(0) = S_0(0)/N = \omega$, by which we infer that

$$\tilde{s}_0(r) = \tilde{s}_0(0)e^{-\alpha r/\sigma} = \omega e^{-\alpha r/\sigma} = \frac{\sigma}{\alpha} f_0(r) \tag{22}$$

where $f_0(r) := \frac{\alpha\omega}{\sigma} e^{-\alpha r/\sigma} = R_0 e^{-R_0 r/\omega}$. Returning to (21) we see that

$$\frac{d\tilde{i}_0}{dr} = \frac{\alpha \tilde{s}_0}{\sigma} - 1 = f_0 - 1.$$

Integrating both sides and using the initial condition $\tilde{i}_0(0) = i_0(0) = \varepsilon$ gives the solution

$$\tilde{i}_0(r) = \varepsilon + F_0(r) - r, \tag{23}$$

where $F_0(r) = \int f_0 = \omega(1 - e^{-R_0 r/\omega})$. See Supp. Fig. 2 for an illustration.

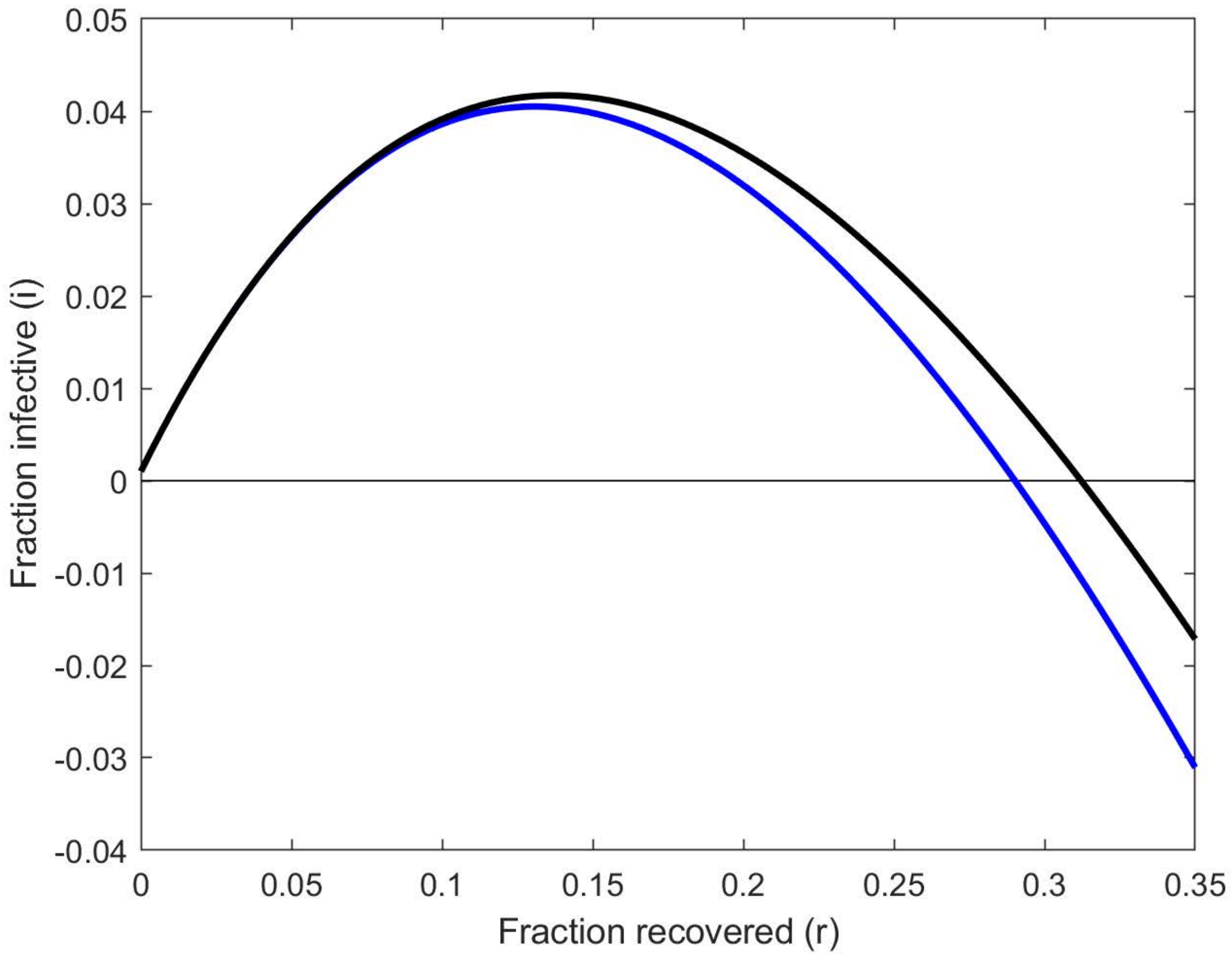

Supp. Fig 2. Graphs of $\tilde{\imath}_0$ and $\tilde{\imath}_1$. The figure shows the solution $\tilde{\imath}_1$ (black) and $\tilde{\imath}_0$ (blue) using the choice of α and ω stipulated by (8) (and values from Figure 1-2 in the main text).

We now solve the equation for $r_0(t)$ in (20), which is separable and can be written

$$\frac{dr_0/dt}{\sigma\tilde{\imath}_0(r_0(t))} = 1.$$

Integrating both sides with respect to t and making the change of variables $x = r_0(t)$ gives

$$\int_0^{r_0(t)} \frac{dx}{\sigma\tilde{\imath}_0(x)} = t.$$

Letting t_0 be the primitive function of $1/(\sigma\tilde{\imath}_0)$ satisfying $t_0(0) = 0$ we thus obtain the implicit solution $t_0(r_0(t)) = t$. Hence, t_0 is also the inverse of r_0 (which exists since $r_0'(t) > 0$ for all $t > 0$), and so

$$r_0(t) = t_0^{-1}(t), \quad t_0(r) := \int_0^r \frac{dx}{\sigma\tilde{\imath}_0(x)}. \tag{24}$$

By differentiating (23) we see that $\tilde{\imath}_0$ is concave. Let r_∞ be the positive solution of the equation $\tilde{\imath}_0(r) = 0$, which thus is unique. By standard calculus this gives rise to a non-integrable singularity in (24), and hence $\lim_{r\to r_\infty} t_0(r) = \infty$. Since r_0 is the inverse of t_0, we see that r_∞ equals the limit $\lim_{t\to\infty} r_0(t)$, known as the final size of the pandemic. We therefore refer to this number simply as $r_0(\infty)$ in what follows. Summing up, t_0 is only defined for values of r in $[0, r_0(\infty))$. Note also that as a

consequence we also obtain

$$\lim_{t\to\infty} i_0(t) = 0 \tag{25}$$

since $i_0(t) = \tilde{\imath}_0(r_0(t))$ so the above limit becomes $\tilde{\imath}_0(r_0(\infty)) = 0$.

Applying the same arguments to (14)–(16) we obtain first a system in terms of normalized variables

$$\begin{cases} s_j' &= -ap_j s_j i \quad 1 \le j \le J \\ i' &= \sum_{j=1}^{J} ap_j s_j i - \sigma i \\ r' &= \sigma i \end{cases} \tag{26}$$

and then an equivalent reduced system of the form

$$\begin{cases} ds_j/dr &= -ap_j s_j/\sigma \quad 1 \le j \le J \\ di_1/dr &= \sum_{j=1}^{J} ap_j s_j/\sigma - 1. \end{cases} \tag{27}$$

Let $(\tilde{s}_1, \dots, \tilde{s}_J, \tilde{\imath}_1)$ be a solution to (27) with $\tilde{\imath}_1(0) = \varepsilon$ and $\tilde{s}_j(0) = S_j(0)/N = w_j$ for $j = 1, \dots, J$. We will write $r_1(t)$ to denote the function described by (24) with $\tilde{\imath}_0$ swapped for $\tilde{\imath}_1$. As in (22)–(23) we obtain the solutions

$$\tilde{s}_j(r) = w_j e^{-ap_j r/\sigma} \quad 1 \le j \le J \tag{28}$$

$$\tilde{\imath}_1(r) = \varepsilon + \sum_{j=1}^{J} w_j (1 - e^{-ap_j r/\sigma}) - r \tag{29}$$

See Supp. Fig. 2 (black curve) for an illustration (using the same values as in Figure 1-2 in the main text). In particular note that it meets the x-axis slightly above 0.3, which coincides with the final size of the pandemic seen for the black graph in Figure 1 (main text).

The philosophy behind formula (8), is that we want to pick the parameters α and ω so that (23) becomes approximately equal to (29). Then, due to (24) we will have $r_1(t)$ approximately equal to $r_0(t)$ and therefore $i_1(r_1(t))$ approximately equal to $i_0(r_0(t))$. Now, by Taylor's formula we have

$$\tilde{\imath}_0(r) = \varepsilon - r + \omega\left(\frac{\alpha}{\sigma} r - \frac{1}{2}\frac{\alpha^2}{\sigma^2} r^2 + O(r^3)\right)$$

and

$$\tilde{\imath}_1(r) = \varepsilon - r + \sum_{j=1}^{J} w_j \left(\frac{ap_j}{\sigma} r - \frac{1}{2} \frac{(ap_j)^2}{\sigma^2} r^2 + O(r^3) \right)$$

where O is the big-O notation of Bachman-Landau. Comparing Taylor coefficients we set $\omega\alpha = \sum_{j=1}^{J} w_j a p_j$ and $\omega\alpha^2 = \sum_{j=1}^{J} w_j (ap_j)^2$ so that the Taylor polynomials of order two of $\tilde{\imath}_0$ and $\tilde{\imath}_1$ coincide. Solving for α and ω gives (8), i.e.

$$\alpha = a \frac{\sum_{j=1}^{J} w_j p_j^2}{\sum_{j=1}^{J} w_j p_j}, \quad \omega = \frac{\left(\sum_{j=1}^{J} w_j p_j\right)^2}{\sum_{j=1}^{J} w_j p_j^2}.$$

Note here that we always have $\omega < 1$. In fact, since $\sum_{j=1}^{J} w_j = 1$ and $0 < w_j, p_j \leq 1$ for all j it follows that $\sum_j w_j p_j \leq 1$, which implies that

$$\sum_{j=1}^{J} w_j p_j^2 > \sum_{j=1}^{J} w_j p_j \geq \left(\sum_{j=1}^{J} w_j p_j \right)^2 \iff 1 > \frac{(\sum_{j=1}^{J} w_j p_j)^2}{\sum_{j=1}^{J} w_j p_j^2} = \omega \tag{30}$$

(with strict inequality since at least one p_j is strictly less than one). These formulas can be put in somewhat neater form by introducing $R_1 = a/\sigma$, i.e. the R_0-value one would have at the beginning of the pandemic if everyone were a super-susceptible. We then get

$$R_0 = \frac{\omega\alpha}{\sigma} = \frac{a \sum_{j=1}^{J} w_j p_j}{\sigma} = R_1 \sum_{j=1}^{J} w_j p_j$$

so $\sum_{j=1}^{J} w_j p_j$ is the reduction-factor due to variable susceptibility. Introducing $f_1(r) = R_1 \sum_{j=1}^{J} w_j p_j e^{-R_1 p_j r}$ and $F_1 = \int f_1 = \sum_{j=1}^{J} w_j (1 - e^{-R_1 p_j r})$, we can write

$$\tilde{\imath}_1(r) = \varepsilon + F_1(r) - r, \tag{31}$$

which should be compared with (23). Note that F_1 is engineered to have same first three Taylor coefficients as F_0. Moreover, introducing

$$t_1(r) := \int_0^r \frac{dx}{\sigma \tilde{\imath}_1(x)}, \tag{32}$$

we obtain r_1 as t_1^{-1} and subsequently $r_1' = \sigma i_1$.

To summarize this section, we argue that if $F_0 \approx F_1$ and $r_0 \approx r_1$, then it should follow that $i_0 \approx i_1$ since $i_j(t) = \tilde{\imath}_j(r_j(t))$, $j = 0, 1$, and $\tilde{\imath}_0$ and $\tilde{\imath}_1$ are given by (23) and

(31), respectively. To formalize this statement a bit, consider now the more general ODE

$$r'(t) = G(r(t), \delta), \quad r(0) = 0, \tag{33}$$

where

$$G(r, \delta) = \sigma(\varepsilon + (1 - \delta)F_0(r) + \delta F_1(r) - r), \quad \delta \in \mathbb{R}.$$

Then $r_0(t)$ is the unique solution to (33) for $\delta = 0$, while $r_1(t)$ is the unique solution to (33) for $\delta = 1$. Since $G(x, \delta)$ is independent of t and continuous in δ for every fixed x, it follows that the solution $r(t) = r(t, \delta)$ of (33) is continuous in δ, uniformly in $t \in [0, T]$ for any $T > 0$, see for example [8] and the references therein. We collect these observations in the following theorem.

Theorem 2.1. *Let $r(t, \delta)$ be the unique solution of (33). Then for any $T > 0$, the solution $r(t, \delta)$ is a continuous function of δ, uniformly in $t \in [0, T]$. Moreover, we have $r(t, 0) = r_0(t)$ and $r(t, 1) = r_1(t)$.*

Our numerical simulations have confirmed that indeed, the difference between i_0 and i_1 is small. To illustrate, we divided the population into 100 (equal size) subgroups (as opposed to 3 in the previous examples), and assigned probabilities according to the formula $p_j = \left(1 - \frac{j-1}{100}\right)^\rho$ where ρ is a parameter to be chosen. This means that we assume a monomial distribution of individual transmission probabilities with exponent ρ. In Supp. Fig. 3 we show the curves for $\rho = 4,\ 2$ and 1 with respective values of ASI (obtained using (8)) equal to 64%, 45% and 25%. Note that basic SIR with ASI becomes a special case of S-SIR if we choose a function p that is either 0 or 1 with a sharp drop at 100ω. These curves are also seen in the embedded pictures.

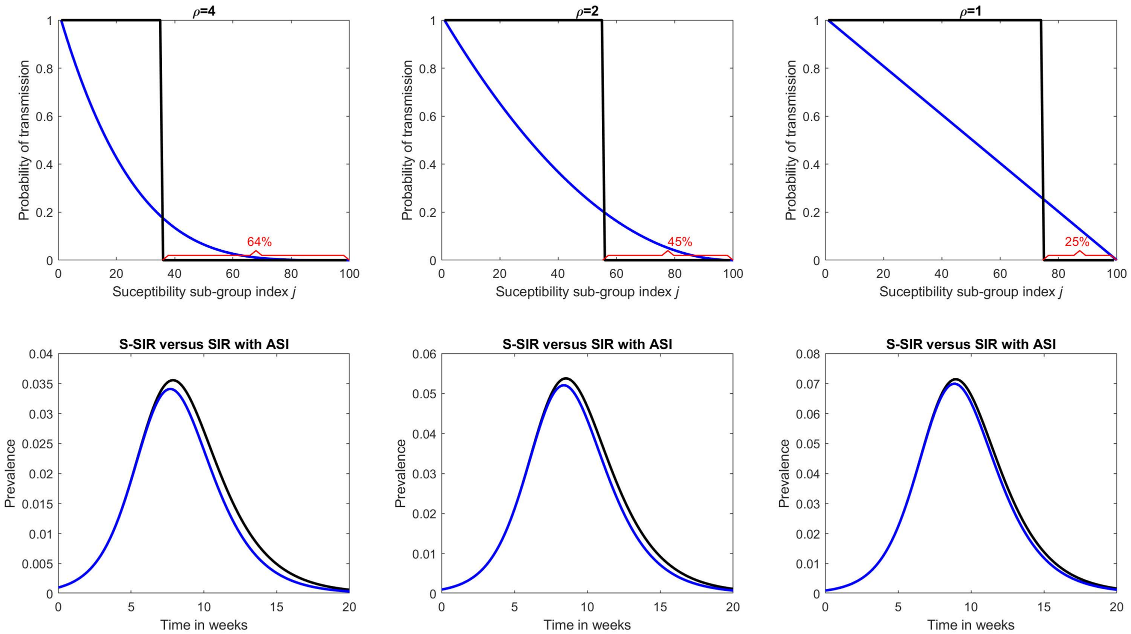

Supp. Fig 3. ***p*-curves.** Top: Curves displaying the probability of transmission when a susceptible person from group j meets an infective. The blue curves correspond to different ρ-values and the black curves display the corresponding binary immunity curves with ASI obtained from (8), yielding 64%, 45% and 25%, respectively. Bottom: corresponding waves of prevalence for S-SIR and SIR with ASI, (using parameters obtained from (8); an even more perfect overlap is possible with free parameters α and ω for SIR with ASI, similar to Figure 1 in the main text.)

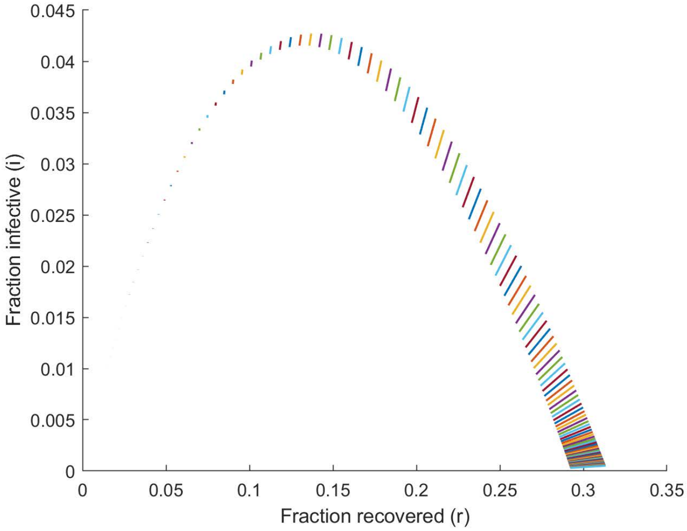

Supp. Fig 4. Envelope foliation. Line between pairs $(r_0(t), i_0(t))$ and $(r_1(t), i_1(t))$ for a grid of t-values, using the same parameters as in Fig. 2 for S-SIR and parameters from (8) for SIR. Note that the inner and outer envelope is given by $\tilde{\imath}_0$ and $\tilde{\imath}_1$ (cf. Fig. 2), and that the slopes are decreasing but positive.

2.1 Further results and a conjecture

It remains to establish proper estimates proving that the resulting solution (i_0, r_0) is near (i_1, r_1). We first establish the following important inequality, which is the explanation behind that the black curve (from S-SIR) always seems to be a bit higher than the blue one (from SIR).

Proposition 2.2. *It holds that $f_1 \geq f_0$. Consequently $F_1 \geq F_0$, $\tilde{\imath}_1 \geq \tilde{\imath}_0$ and $d\tilde{\imath}_1/dr \geq d\tilde{\imath}_0/dr$.*

Proof. The choice of ω and α is designed so that $f_1(0) = f_0(0)$ and $f_1'(0) = f_0'(0)$. Moreover we have

$$f_1(r) - f_0(r) = \frac{1}{\sigma}\left(\sum_{j=1}^{J} w_j a p_j e^{-ap_j r/\sigma} - \omega\alpha e^{-\alpha r/\sigma}\right).$$

Multiplying with $e^{\alpha r/\sigma}$ we obtain $\sum_{j=1}^{J} w_j a p_j e^{(\alpha - ap_j)r/\sigma} - \omega\alpha$ which is a strictly convex function (unless $\alpha = ap_j$ for all j, which means that all probabilities are the same and the model collapses to the standard SIR model, and there is nothing to prove) that in addition equals zero and has derivative zero at $r = 0$ by construction. By convexity it follows that the function is non-negative, giving $d\tilde{\imath}_1/dr \geq d\tilde{\imath}_0/dr$ with strict inequality for $r > 0$. Convexity also implies that the function and its derivative are increasing. The desired inequality follows by integrating $d\tilde{\imath}_1/dr \geq d\tilde{\imath}_0/dr$, keeping in mind that $\tilde{\imath}_1(0) = \tilde{\imath}_0(0)$. □

Proposition 2.3. *Let r_0 be the solution to* (20) *and r_1 the solution to* (26)*. Then $r_1 \geq r_0$.*

Proof. Let $g(x) = \sigma\tilde{\imath}_0(x)$. Then $r_0'(t) = g(r_0(t))$ while $r_1'(t) \geq g(r_1(t))$ by Proposition 2.2. Since $r_1(0) = r_0(0)$, standard theory of ordinary differential equations then dictates that $r_1(t) \geq r_0(t)$ for $t \geq 0$. □

However, we believe there is more to say than what is captured in the above two

propositions. Supp. Fig. 4 gives an illustration of how the pairs $(i_0(t), r_0(t))$ and $(i_1(t), r_1(t))$ evolve with time. Note how the distance seems to be gradually increasing and always points in the north-east direction. We have not been able to prove this nor the closely related following observation, which in a more concrete way would imply that i_1 and i_0 are close.

Conjecture 2.4. *We have $i_1 \geq i_0$ and furthermore, $s_0 - \sum_{j=1}^{J} s_j$ is an increasing function.*

To understand the implications of the conjecture, recall that the final size of the pandemic solves $\tilde{i}_j(r_j(\infty)) = 0$, $j = 1, 2$, and that these two points tend to be quite close, see for example Supp. Fig. 2 where the difference $r_1(\infty) - r_0(\infty)$ is around 0.02 or 2%. Since $s_0 - \sum_{j=1}^{J} s_j = \omega - 1 + (i_1 + r_1) - (i_0 + r_0)$ and $r_1(\infty) - r_0(\infty)$ is the limit of $(i_1 + r_1) - (i_0 + r_0)$ as $t \to \infty$, the conjecture implies that both the difference $r_1(t) - r_0(t)$ as well as $i_1(t) - i_0(t)$ are bounded by $r_1(\infty) - r_0(\infty)$.

References

1. Hickson R, Roberts M. How population heterogeneity in susceptibility and infectivity influences epidemic dynamics. Journal of Theoretical Biology. 2014;350:70–80.
2. Miller JC. A note on the derivation of epidemic final sizes. Bulletin of mathematical biology. 2012;74(9):2125–2141.
3. Jones TC, Biele G, M¨uhlemann B, Veith T, Schneider J, Beheim-Schwarzbach J, et al. Estimating infectiousness throughout SARS-CoV-2 infection course. Science. 2021.
4. Yang Q, Saldi TK, Gonzales PK, Lasda E, Decker CJ, Tat KL, et al. Just 2% of SARS-CoV-2- positive individuals carry 90% of the virus circulating in communities. Proceedings of the National Academy of Sciences. 2021;118(21).
5. Diekmann O, Heesterbeek H, Britton T. Mathematical tools for understanding infectious disease dynamics. In: Mathematical Tools for Understanding Infectious Disease Dynamics. Princeton University Press; 2012.
6. Brauer F. The final size of a serious epidemic. Bulletin of mathematical biology. 2019;81(3):869–877.
7. Brauer F, Castillo-Chavez C, Feng Z. Mathematical models in Epidemiology. Springer; 2019.
8. Artstein Z. Continuous dependence on parameters: On the best possible results. Journal of Differential Equations. 1975;19(2):214–225.
