# Supplementary material for: A note on variable susceptibility, the herd-immunity threshold and modeling of infectious diseases
Source: PLoS One. 2023 Feb 15;18(2):e0279454. doi: 10.1371/journal.pone.0279454 (PMC9931097; doi:10.1371/journal.pone.0279454)
Supplement: Attachment — Submitted filename: Report_PONE-D-22-06269.pdf [file pone.0279454.s004.pdf]

Report on (Ref. PONE-D-22-06269)
A note on variable susceptibility, the herd-immunity threshold and modeling of infectious diseases

The authors have satisfied almost all of the comments from the original submission. I have few more comments in different dimension about this new version is that the variable S and I including few numerical results are hard to follow.

Comments on R1 version:

1. For multiple demonstration of SIR model, it's really not clear to me why variable susceptibility; does it mean the S class is depending on time periodic parameter $\nu(t)$ or discrete case of S-SIR model. Please clarify.

2. In all figures, use the readers friendly legend/caption/title, for example '**prevalence**', instantly it is very hard to realize.

3. In many figures, there is no horizontal or/and vertical notion over the figure. Check Supplementary Fig. 2-4. Please revise.

4. The introduction is revised properly. However, to improve the literature, see the following articles where the authors' used hybrid techniques of modeling:
Coronavirus outbreak and the mathematical growth map of Covid-19, Annual Research & Review in Biology, 2020
Mathematical Modeling and Covid-19 Forecast in Texas, USA: a prediction model analysis and the probability of disease outbreak, Disaster Medicine and Public Health Preparedness, 2021.
Vaccine efficacy and SARS-CoV-2 control in California and U.S. during the session 2020–2026: A modeling study, Infectious Disease Modelling, 2022.

5. In section 1.1 Contemporary models for COVID-19, based on citations and prevalence percentage, it is claimed that SIR and SEIR will produce the similar results. It means E class plays minor roles. How? based on the present Fig. 3. Please check first:
Dynamics of SEIR Model: A case study of COVID-19 in Italy, Results in Control and Optimization, 2022.
Consider $R_0 < 1$ and show one more figure for visible results of SEIR model compared with the present study and I believe it will accept and increase the accuracy of this study.

6. There are punctuation errors, please check the manuscript carefully.

The reviewer is convinced and satisfied with the methodology, model hypothesis and analysis techniques used in this study and expecting the proper clarification of the above.

Finally, after satisfactory explanation and modified revision in the discussion, it is recommended to publish the article.
