# Supplementary material for: A note on variable susceptibility, the herd-immunity threshold and modeling of infectious diseases
Source: PLoS One. 2023 Feb 15;18(2):e0279454. doi: 10.1371/journal.pone.0279454 (PMC9931097; doi:10.1371/journal.pone.0279454)
Supplement: Attachment — Submitted filename: Response to the reviewers.docx [file pone.0279454.s003.docx]

Rebuttal letter

We are very grateful for the opportunity to do a major review and address the concerns raised by the reviewers. We first list the major changes:

1. The previous focus on whether other models for predicting SARS-CoV-2 were accurate or not seems to have shifted focus away from the key contributios of our paper, which is of relevance for modeling any infectious disease. Consequently, we have removed much of this discussion, and what remains has been rephrased much less assertive.
2. Similarly, the focus on pre-immunity is misleading since the results are of relevance still today, about how to model SARS-CoV-2 in the presense of all sorts of vaccine induced immunity and waning immunity from previous strains. We therefore refer to this parameter as ”artificial sterilizing immunity” (ASI) rather than ”population pre-immunity”.
3. Due to points 1 and 2, the introduction as well as large parts of section 1 have been completely rewritten (see text in red in the extended version).

The main finding of the paper is that compartmental models, taking various heterogeneities into account, behave just like a standard SIR if we incorporate artificial steralizing immunity. Once this is established, we derive actual formulas for the Herd-Immunity Threshold in terms of the parameters of the simplified model. It seems that we have failed to convey this message in the first version. The key result was *not* to demonstrate numerically that variable infectivity is not important, whereas varaible susceptibility lowers the overall curves, which has been shown before in various publications, as pointed out by Reviewer 1. We have tried to highlight this by

4. Better explaining what is meant by Herd-Immunity and R0. We also added several references on this topic.
5. Clearly stating that while the general pattern was known (we thank reviewer 1 for useful references), our main contribution is to demostrate this phenomenon mathematically and also to quantify it in terms of actual formulas involving few parameters that actually can be estimated. This has not been done before.
6. We show numerically that these findings seem to extend also to more intricate models where various other heterogeneities are incorporated. To illustrate this, there is now a new section 4 (previously section 7 in the supplementary material) which shows that it is possible to accurately match the curves by Britton et al, published in Science 2020, using ”our” model SIR with ASI.
7. SIR with ASI is indeed over-simplified, but this is the whole point. While models of the type used by Britton et al (and others we refer to) are full of constants whose values are impossible to estimate with any accuracy, the output is the same as SIR with ASI, and the parameters in this model can more easily be estimated from real time series which, at least in theory, should simplify for predictions of the Herd Immunity Threshold and other key parameters.
8. We added a small section 2.2 noting that the final size of the pandemic can also be predicted if the ASI-level is known.

Due to the above points, there are major changes also in the remaining sections 2-3 (all in red, especially in section 2.1 where R0 and HIT are discussed). In addition we have omitted most of the first section of the Supplementary Material, which mainly consisted of examples for how to properly build the equation system. While this may be instructive for some readers, it had a bit of a schoolbook aire and most likely readers who are interested in the supplementary material are already well acquaintanced with basic modeling.

We hope that you find the new version more balanced and to the point.

Response to the editor (and reviewer 2)

The comments by reviewer 2 were not attached, and we have reached out to the journal several times about this. Since today is the deadline, we submit a revised version without having seen the comments of reviewer 2. We would of course be happy to take these comments into account in another round of revision, if we could get them.

Judging from the remarks of the editor, there were some questions about sero-conversion, the definition about herd-immunity, and the fact that the model is overly simplified. The latter two points are addressed in points 4 and 7 above. Concerning sero-conversion, this is really beside the main point of the paper. The discussion about whether various models used initially to predict massive society spread were accurate or not, is now limited to Section 1.2. For this topic, it is of course of relevance how the sero-prevalence is measured. As far as we understand, sero-prevalence is a good indicator of how many have had SARS-CoV-2 in the short term, and we added references backing this up [19-21]. The data of sero-prevalence levels in Sweden comes from the Public Health Authority which used thousands of samples from blood samples collected during general practice in the Swedish Health Care system. We find no reason to assume that this data should not be reliable. Of course, once we get the comments by reviewer 2, we will be able to address this issue more in detail.

Finally, concerning naming of figures, we did our best to follow the guidelines and hope the new naming system is in line with the journal style.

Response to reviewer 1 (reviewer comments in red)

The essence of the paper is a mathematical result which shows that a compartement model in which susceptibility varies between individuals, behaves the same as a much simpler model in which an artificial level of immunity is included, even if no individual is immune in a strict sense. This result is new and has not been proved or even hinted at in any of the references suggested, and hence we maintain that our contribution is original and of interest to the modeling community. We understand that the discussion about Imperial College and predictions about COVID19 took the focus from this, and have tried to rewrite accordingly. Indeed, we do not want to be authors of an ”opinion piece dressed as science”.

I have many concerns about this paper. Most fundamentally, I feel the authors do not demonstrate a deep understanding of the state of the art in mathematical modeling. So it makes me very uncomfortable that there is a thread running through this paper and the other papers of their series in which they criticize modelers, and imply that they have solved everything.

We are grateful that the reviewer shares his feelings about the text. It has certainly not been our intention to criticize “all modelers”, nor would it be very constructive to do so, so we have done our best to rewrite accordingly. On the other hand, none of the concerns raised pertain to the actual findings in the paper, whereby we insist that the paper is scientifically sound and that the results are original as well as interesting.

We here briefly discuss some other influential contributions, to highlight what distinguishes our paper. A common trend in epidemiological modeling is to make models with many compartments in order to more accurately reflect the complexity of society. However, with many compartments comes many constants for how the disease spreads between these compartments, and reliable estimation of these constants quickly becomes impossible. One way to deal with this is to make educated guesses and run the model for various choices, in which case the model serves to illustrate trends of how different types of heterogeneities affect output, but not for actual prediction of spread. An example of this type is the Science-paper by Britton et al cited below, which basically concludes that population heterogeneity lowers the Herd Immunity Threshold, but says nothing concrete about the magnitude. However, even in their most heterogeneous scenario, the final size of the pandemic is reached within months and is 35%, which is very far from what actually happened in Sweden, a fact which to our knowledge neither Britton nor anyone else has tried to explain.

A paper where they use state of the art estimates of parameter values to manually determine all involved constants is Sjödin et al, “COVID-19 demand and mortality in Sweden...”, published in Int. Jour. Epidemiology, where they use a model with 98 compartments and manage to fit almost perfectly ICU-occupancy and deaths. However, the curve of total recovered levelled out at 30% at a time point when it was well known that only around 10% had had COVID-19, and hence the model had nothing to do with reality, which seems to have not bothered neither the journal nor the authors. In all papers we have examined, including those the reviewer suggests below, the model predicts one major wave (assuming, as in Sweden, that interventions are fixed) with magnitude much higher than was observed in Sweden. We have not seen any paper addressing this issue, or even recognizing it. Apparently, this issue has given our paper an air of arrogance, which is unfortunate.

Having said all this, we have tried to remove most of the discussion about what happened in Sweden to another paper which deals exclusively with this topic, and refrain from claims that other models do not work, in order to focus on our key contribution in this paper. Our paper exposes mechanisms that could partially explain the above dilemma, but even if one does not believe that state of the art models overestimate the spread, this result is interesting in its own right.

Our work stands out in that it takes a complicated model and replaces it with a simple. We prove mathematically that a multicompartment model with variable susceptibility (including many constants no-one will ever be able to reliably estimate) yields almost the same output as a simple model with an artificial immunity built into it (including few constants which are more realistic to estimate). This observation is highly relevant also for simplifying present modeling, since there are all sorts of waning partial immunity in society by now, from vaccines as well as previous infections with different variants. In addition, we show that models such as those published in Science by Britton et al can be simplified analogously without any loss of accuracy (see Section 3 in the revised version).

Major concerns:


1) "it became clear that the models were overly pessimistic." This statement in the abstract makes me worry that this is actually highly motivated reasoning masquerading as science. It is good that the authors recognize this claim is not justified - lines 6-7 and 12 of the text contradict the claim in the abstract that this was clear, and acknowledge that no such claim can be made. But now I'm wondering why did they make a claim that they knew was false in the abstract? As a result, at this stage I have significant doubts that I'm about to read a scientific paper. I've read plenty of opinion pieces dressed up in academic language during the pandemic, and I don't want to read one in a legitimate scientific journal.

As mentioned above, this issue really diverts attention from our key findings, so we have removed most comments of the above type from the text. One can make a pretty strong case that the Imperial College model was overly pessimistic, by comparing with Sweden, and a discussion about this is still present in Section 1.2, where we are much more humble than in the first version. We hope the reviewer is comfortable with the new wording.
Related to this, the authors need to get rid of the judgmental statements about existing models and modelers throughout this and the other papers in this "series of articles aimed at better understanding the discrepancy between model prediction and observed data." It is not clear if they actually know the structure of the model whose implied failure is used to motivate the study - someone reading this paper's description would assume that Imperial used a differential-equations based SEIR model that assumed a well-mixed population. That is not what they did.

It was certainly not our intention to insult their very impressive work, which based on the knowledge at hand 2020 was reasonable and probably the best one could do at the time. In retrospective it is still important to try to evaluate accuracy and what could have been missed or improved, which is our intention. We thank the reviewer for pointing out how it sounded and we have removed judgmental statements.
2) Far too much of this paper is saying "we show in [5] that this cannot be explained without pre-immunity". [5] is a preprint, and I am uncomfortable relying so strongly on results that have not undergone peer review.

Point taken, we removed most of the discussion of what is in our other papers so that the paper stands on its own.

3) "The NPI's have remained virtually constant throughout the cold period of 2020-2021... in theory means that the mathematical models should work, just with a new R_0-value adopted to the new situation. However in [5] we make a major effort to produce refinements of state of the art compartmental models, taking various sorts of population heterogeneities into account [and can only fit data by assuming 60% are immune]".

I'm not in a position to comment on the NPIs in Sweden, but when I look at mobility data for Sweden, I see that mobility was highly variable, and that there was a very significant drop in average movement during the second wave, and the drop is likely not uniform in time. Although I have not read [5] nearly as closely as this draft, the claim is made that we can treat behavior as constant because the NPIs are constant. But the measured behavior was not constant, so I think the claim that this can be ignored is false (this is part of the reason I am uncomfortable with such high dependence on a preprint). Additionally, the fact that the behavior changeis likely bimodal (with some individuals significantly reducing movement while others continue as before) is very likely to result in a subpopulation with significantly reduced probability of infection - which will be indistinguishable from a subpopulation having pre-existing immunity.

All the above points are correct and treated with care in [5], where mobility charts from google are present. Mobility is variable, but it does not vary in any way that can explain the rise and fall of infection. Thus, if anything, this points to variations in mobility (as measured by google) having limited bearing on disease spread. Moreover, a subpopulation isolating can clearly not be distinguished from a subpopulation with pre-immunity, but it is certainly unreasonable to assume that 60% have the ability to isolate when schools are open and facemask use is minimal. Having said that, this discussion belongs to [5] so we removed this part from the present manuscript.
4) lines 38-40 "In this article we show mathematically that, rather surprisingly, variatio0ns in infectivity has no bearing on the model curves, whereas variations in susceptiblity manifests itself as pre-immunity on the macro level. "

And lines 167-173:
"Incidentally, at the end of each paper [10-12], Kermack and McKendrick stress that a weakness in their model is that they assume uniform susceptibility, which they consider unrealistic in many cases. However, it seems that they never got around to correct this issue, and we have not found a rigorous analysis of how to model this mathematically elsewhere in the literature either. In particular, the formula 1 -1/R0 for the Herd-Immunity Threshold (HIT) (see SM Sec. 4), which stems from their seminal papers, may very well be inaccurate. In the coming section we derive a refined version of this formula taking variable susceptibility into account."


The result of variations in infectivity is not a surprise to me. The variation in infectivity is unimportant under specific circumstances, which do apply in a well-mixed compartmental model settings (and in disease spread in some random networks as well). The reason for this is straightforward: the model assumes that the number of infections is large enough for a law of large numbers to apply (otherwise the model would not be deterministic). As such we can safely average the number of infections caused in a well-mixed population over all individuals. Assuming that infectiousness and susceptibility are uncorrelated, the average infectiousness is the same early or late in the epidemic. (this breaks down if they are biologically correlated or if the higher infectiousness is due to behaviors that also increase susceptibility, in which case the more susceptible are infected sooner on average).

We thank the reviewer for pointing out that these results are not surprising, and can be deduced by heuristic arguments. We have incorporated this in the new version, as well as many of the references suggested by the reviewer, and hope that this has helped to clarify what distinguishes our work from those references.

[note, if a population is made up of many small groups such as households, then superspreading does have an impact, but this model does not contain households. If the model is intended to predict probability of establishment, then variation is infectiousness (but not susceptibility) matters]

We agree to these points but our paper is not about these issues.
As for variation in susceptibility, the disease preferentially removes the highly susceptible, leaving a less susceptible residual population. There is a large body of work that analyzes this effect. I am surprised that the authors were unable to find a rigorous analysis of this. I would start with:

The references below discuss variations in susceptibility to various degrees, and reach the conclusion that such variations limit spread. The fact that the highly susceptibles are infected early and that the spread then slows down, is no surprise. What is surprising is that once spread stops among the highly susceptible, it also stops in the other compartments, which makes the wave of infections look precisely the same even with very different model parameters. This is our main discovery, and the underlying reason why a simple SEIR with “artificial immunity” always does an equivalent job as a multicompartment SEIR with variable susceptibility. The papers below do not contain this finding, but we are grateful that the reviewer pointed them out so that we can provide a more complete bibliography.
- Britton, T., Ball, F. & Trapman, P. A mathematical model reveals the influence of population heterogeneity on herd immunity to SARSCoV-2.

This paper does not include variation in susceptibility. A follow up preprint that was never published (main conclusion was that it is easier to acquire herd immunity by natural infection than by vaccination) did include variations in susceptibility, and also had a remark that variations in infectivity had no bearing on model curves, as we prove here more rigorously.

- Gou, W. & Jin, Z. How heterogeneous susceptibility and recovery rates affect the spread of epidemics on networks.

The models in this prepandemic paper (which is cited by 15), does contain variations in susceptibility in a manner analogous to our model and the one by Britton et al. They derive a number of formulas for R0 and the final size of the pandemic, in terms of the constants involved in their models. This is not very difficult and also not very informative. The numerical section has graphs showing how correlations between their various types of compartments affect R0 and final size, which is interesting for understanding the dynamics, but not very helpful for accurate modeling. Moreover, the way the formulas are deduced leave doubt as to whether the authors understand mathematics well. For instance, they seem to believe that a decreasing differentiable function bounded below by 0 must have a derivative that goes to 0, which clearly is false (see comments following (3.5)). We have decided not to cite it.

-Gerasimov, A., Lebedev, G., Lebedev, M. & Semenycheva, I. COVID-19 dynamics: A heterogeneous model.

This paper builds a model only focusing on variation in susceptibility, basically the same model as we use in our work, and by testing they find that heterogeneity in susceptibility lowers the overall incidence compared with a homogenous model. This is not difficult to do, and their results do not overlap with our main findings.
-Hickson, R. & Roberts, M. How population heterogeneity in susceptibility and infectivity influences epidemic dynamics.

This pre-pandemic paper has 27 citations, and includes a model similar to ours. It contains some original mathematics on how to solve the equation system, although it is unclear to us why this is needed since the equations can be solved in matlab without introducing an orthogonal polynomial basis. The remainder of the paper deals with numerical tests of their model. However, in contrast to our findings and the intuition of the reviewer, as well as all papers above and below, they find that variability in susceptibility has no effect on the output.
-Dolbeault, J. & Turinici, G. Social heterogeneity and the COVID19 lockdown in a multi-group SEIR model.

This paper contains a two group heterogeneous model and is mainly concerned with what happens if a small part of the population do not follow lock down social distancing. They thus try to model the same as Britton et al above, but interestingly their model is fundamentally different, and coincides instead with our model for variable susceptibility. The reason for this is that the authors consider that socially active people are more likely to get COVID, without incorporating that they are also more likely to spread COVID. A discussion of how to accurately model variations in social behavior goes beyond the scope of our article, so we will not cite this contribution.
-Miller, J. C. Epidemic size and probability in populations with heterogeneous infectivity and susceptibility

This paper contains modeling on networks and contain some quite heavy machinery, for which one is directed to previous papers on this type of modeling. While it is unclear how much the network affects the conclusions regarding variations in susceptibility, the overall conclusion seems to be that heterogeneity lowers the chance of an outbreak as well as final size of the pandemic if an outbreak occurs. We agree to this conclusion and cite it. Again, our work establishes much more than this.
-Miller, J. C. Bounding the size and probability of epidemics on networks

Google scholar does not seem to find this paper. However, since again it is about modeling on networks and targets size and probability of outbreaks, it is not likely to have any overlap with our findings.
-Miller, J. A note on the derivation of epidemic final sizes
Really nice paper about how to derive equations for the final size of the pandemic in various probabilistic models, thank you for pointing this one out! It aligns very well with our findings, and we added a Section (#2.2) describing how to derive the final size of the epidemic within our framework.

The overlap with our key findings is however minimal. We followed some of the references as well to really make sure our findings are original and new, for example Katriel “the size of epidemics in populations with heterogeneous susceptibility”, but the conclusion is the same; While the results seem both correct, informative and highly non-trivial from a mathematical perspective, the overlap with our paper is minimal. Our paper is not about predicting the final size of pandemics nor does it try to simply establish that variable susceptibility lowers it, *our paper is about how to actually model spread with an unknown distribution of variable susceptibility, and provide accurate formulas for the HIT, not only noting that it seems to be lower, either based on heuristics or numerical testing.*
This last one does a lot to explain the issues that the authors raise (heterogeneity in susceptibility mattering but not infectiousness). It also does age-structured models and many other cases. I think it only touches on the final size relations, though likely the arguments used can apply for intermediate times.

There is some subtlety regarding the herd immunity threshold that has not been studied extensively previously. It depends on whether the immunity is achieved through infection or through random vaccination - for the result on infection acquired immunity I would read Gabriela Gomes & colleagues recent papers/preprints. For random vaccination it remains 1-1/R_0.
Thank your for pointing this out, this is a very important point. We added a discussion about this in 2.1 and also SM 1.2, where we prove the details. Note that Britton et al has a preprint with the same message. Both Britton and Gomes et al demonstrate this by building simple ”toy” models (2 compartments for susceptibility in the case of Gomes) and then observe this phenomenon by testing their model. Our paper is more rigid in that prove that this effect is indeed there in a multicompartment susceptibility stratified SIR, and even give a formula for the new HIT.

5) I am concerned by the discussion starting at line 55:
"For example, a SIR-model has no memory, i.e. it does not keep track of how long a person is sick, but the original equation systems in [10] did. However, it has been shown that this, as well as other factors such as randomness has a very limited bearing on the model curves, see e.g. [13]"

Memory may be important in the initial stages but has no bearing for major outbreaks, which is what our paper studies. However, a discussion of this is completely off track so we have removed this comment.

Given that the paper starts by referring to the Imperial modeling work and focuses significant attention on implied failure of their predictions, this statement should not be made because it seems to imply that Imperial's (and other models) use a model as in the SIR model described. Most state of the art models (including Imperial's) do have memory.
Thank you for pointing this out, we have removed this comment. However, the Imperial Report 9 did not come along with a supplementary material where one could see the code, but for example the later Science paper by Walker et al ”The impact of covid 19 strategies for mitigation and suppression in low and middle income countries”, with many authors same as the authors of report 9, does have a detailed SM and they did not bother to include memory. Indeed, why should they since everybody seems to agree that this has no bearing under a major outbreak.
I don't know that the results from [13] are consistent with this claim. It's not clear what is meant by 'randomness', but definitely some forms of randomness matter and the details of the generation interval plays an important role in the early dynamics.
We agree with this statement and apologize for not having been more clear about discussing a major outbreak. In any case, the comment has now been removed.

6) It is not clear to me how system (3) was handled in terms of R_0. Was R_0 defined initially and then a subset treated as immune (so the effective reproduction number drops)? This is what the statement seems to imply. Or was R_0 defined based on the population after the subset was made immune. In either case, this is just the usual SIR model but confined to a smaller population.
Equation (3) was simply stating initial conditions. The formula for R0 in the model with preimmunity was given in (7), from which it directly follows that the answer is the second option, so that R0 actually determines the speed at which the system grows when the disease is introduced. This value of R0 is (more or less) the same as one would estimate using e.g. EpiEstim from a time series of the initial phase from the model. We have added this comment and discuss it extensively in the new version of Section 2.1. We hope this clears up any confusion.

This is also visible for example in Fig 1 as the curves for I start out identically, indicating that each different model is based on the same actual R0 number. This is an important point which we have tried to further clarify: Fig 1 and 2 do not show that ”if one lowers susceptibility in one group, the incidence, HIT etc goes down” which is pretty obvious, but rather ”if there is variable suseptibility and one *measures* R0 at the onset, then the incidence will peak lower and decay much faster, than what a homogenous model would predict”. This is an interesting result, which we have tried to highlight.


Minor:

1) A source is needed for this claim: "most research teams use extensions of SEIR for modeling COVID-19" Additionally the comment needs to distinguish between an SEIR model in terms of progression of infection and the differential equations SEIR model. E.g., the Imperial Model that the authors seem to focus on does not use an SEIR compartmental model, though they assume SEIR progression of infection. Their model is an individual based simulation. It has households, workplaces, ...
The claim is made based on reading lots of papers, and clearly it is impossible to find a source for such a claim. We have removed it.
2) I don't believe I have encountered the word "fictive" before (google tells me what it is, but I think it is not standard English).

3) It's not appropriate to refer to the epidemic curves as "bell-shaped". That usually implies e^{-x^2} type behavior. These have exponential growth early and exponential decay late.
e^{-x^2} is referred to as the Gaussian by most mathematicians, whereas bell shaped is more general. Also the Gaussian has super-exponential decay/growth, since clearly it decays much faster than e^{-ax} for some fixed a as x->infty. Anyways, we reformulated this part.
4) To my eye, most predicted epidemic curves are not symmetric.
Ok, we have removed this comment.
5) caption for fig 1: the "second two" do not level out below the HIT. They level out below the HIT predicted by the "standard" model. But that's because they use they are created using a different model. They overshoot the HIT (the HIT is not defined based on a specific canonical model, it's defined based on when R_e=1 for the actual system). Please introduce a notation for the HIT based on the classical model, and discuss it. The discussion of HIT in the context of a different model is confusing because I keep assuming we are discussing the HIT of that model.

The figure text clearly speak of the “*classical* HIT”. The text also makes the distinction between HIT and *classical* HIT, (defined by 1-1/R0). There are in fact various ways to define the HIT, discussed in Fine P, Eames K, Heymann DL. “Herd immunity”: a rough guide. Clinical infectious diseases. 2011;52(7):911–916., which we now cite. It is of course pivotal that our definition of HIT is clear for the reader, so therefor we have rewritten Section 2.1, (our definition is the same as suggested by the reviewer, which has also been clarified).

We agree that both R0 and HIT are model dependent. Hence, it is quite plausible that the formula HIT=1-1/R0 would be valid in general, and not only for the most basic SIR model. In fact, Britton et al’s paper is entirely about showing that this formula does not hold and that the HIT can be substantially lower than 60%, even if R0=2.5 (which gives 0.6=1-1/2.5). If this was well known, why did Science publish it?

In any case, Britton et al do not provide any formulas, they just make a numerical observation after constructing a model and running it. Our contribution is that 1) a variable susceptibility model can be reduced to a simpler pre-immunity model, and we give a formula for the correct R0 which describes the speed of increase when the disease is novel, in this simplified model, (see the answer to 6) above.) 2) In the simpler model, we provide a formula for HIT which is different than the *classical* formula 1-1/R0, which hence may be misleading. Section 2.1 concluded with saying that the *classical* formula is inaccurate, not that the model somehow miraculously levels out below its own HIT. These issues are highlighted in the new version, and the adjusted formula for HIT is now formula (2), whereas the classical formula is formula (1).

6) It is not clear to me why hard-hit towns in Italy which underwent some of the strictest NPIs anywhere would be expected to have high seroprevalence.

We agree to this, our phrase was misleading. The claim by the authors we cite is that the relatively low level of seroprevalence provides unexpectedly large protection against new outbreaks. To this one can add other point observations such as around 20% of hospital staff in major Swedish hospitals, who did not use masks, becoming infected during the first wave. Anyways, this discussion is beside the point and has been omitted.
7) I am not aware of any serious academic study of "immunological dark matter".

See Karl Friston, Anthony Costello, and Deenan Pillay. Dark matter, second waves and epidemiological modelling. BMJ Global Health, 5(12):e003978, 2020.
