# Supplementary material for: A note on variable susceptibility, the herd-immunity threshold and modeling of infectious diseases
Source: PLoS One. 2023 Feb 15;18(2):e0279454. doi: 10.1371/journal.pone.0279454 (PMC9931097; doi:10.1371/journal.pone.0279454)
Supplement: Attachment — Submitted filename: Response to the reviewers 2.docx [file pone.0279454.s005.docx]

Response to the reviewers.

We thank the reviewer for insightful comments that helped improve the manuscript. We have revisited the entire text, rewriting bits and pieces in order to make it more readable and understandable. We have taken particular care to figure axes labels, figure titles and figure texts.

All major changes are marked in red in the manuscript titled “with track changes”. Note that text that was slightly rewritten was also marked in red, so there are no essentially new parts.

To address the specific questions by the reviewer, we have clarified that “variable susceptibility” refers to differences between individuals in the probability to get infected, given a meeting with an infective person, and not, as one could get the impression, individual variations in susceptibility over time. We have clarified this in the introduction. We added a headline “novel contributions” to the introduction in order to make this more clear to the reader. The above adresses comments 1-4 by the reviewer. Concerning 5, we have included a number of relevant new citations, among them three of those that the reviewer suggested. We also added newer research indicating that variable susceptibility indeed was present at the onset of the pandemic, see reference [18] Kundu et al, Nature Communications. Also reference [4] by Carlsson and Söderberg-Naucler has in the meanwhile gotten published, so we put more emphasis on this in the discussion on the discrepancy between model output and reality.

Concerning 5, yes we claim that E plays a minor role, as long as parameter values are free, in the sense that for each parameter configuration for SEIR we can get an almost identical curve using SIR. This is demonstrated in Fig. 3, but the conclusion is well tested on a number of different parameter settings, and this is discussed in depth in Section 4.1. For clarity, these findings have been highlighted also in 2.1, see the new text in red.

Finally, we refrain from adding new graps related to R_0<1, since we feel this would make the manuscript too long and deviate attention from the key findings. Concerning 6, we have carefully read the entire text with a focus on English and punctuation.

Sincerely,

Marcus Carlsson
